# Independent evolution toward larger body size in the distinctive Faroe Island mice

**DOI:** 10.1093/g3journal/jkaa051

**Published:** 2021-01-04

**Authors:** Ricardo Wilches, William H Beluch, Ellen McConnell, Diethard Tautz, Yingguang Frank Chan

**Affiliations:** 1 Friedrich Miescher Laboratory of the Max Planck Society, 72076 Tübingen, Germany; 2 Max Planck Institute for Evolutionary Biology, Department of Evolutionary Genetics, 24306 Plön, Germany

**Keywords:** genetic mapping, evolution, mouse, QTL, island mice

## Abstract

Most phenotypic traits in nature involve the collective action of many genes. Traits that evolve repeatedly are particularly useful for understanding how selection may act on changing trait values. In mice, large body size has evolved repeatedly on islands and under artificial selection in the laboratory. Identifying the loci and genes involved in this process may shed light on the evolution of complex, polygenic traits. Here, we have mapped the genetic basis of body size variation by making a genetic cross between mice from the Faroe Islands, which are among the largest and most distinctive natural populations of mice in the world, and a laboratory mouse strain selected for small body size, SM/J. Using this F2 intercross of 841 animals, we have identified 111 loci controlling various aspects of body size, weight and growth hormone levels. By comparing against other studies, including the use of a joint meta-analysis, we found that the loci involved in the evolution of large size in the Faroese mice were largely independent from those of a different island population or other laboratory strains. We hypothesize that colonization bottleneck, historical hybridization, or the redundancy between multiple loci have resulted in the Faroese mice achieving an outwardly similar phenotype through a distinct evolutionary path.

## Introduction

Discovering the genetic basis of naturally occurring phenotypic variation is an important step toward understanding how traits change through time. Knowing which genes are involved in controlling traits may allow inference of how selection operates in shaping organisms and help to explain natural phenomena like rapid evolution (Barton and Keightley 2002). Selection is a directional process, which means that unlike neutral processes like drift, similar selection pressures tend to produce similar phenotypic outcomes across independent replicates. This phenomenon, known as parallel evolution, is often considered as indicative of selection (Schluter *et al.* 2004). Equally, mutation biases, or other environmental induced responses may also repeatedly produce similar outcomes. By studying parallel evolving systems, we may gain insight into the relative importance of selection and chance (Conte *et al.* 2015). Despite many remarkable studies identifying the genes associated with parallel phenotypic changes (Chan *et al.* 2010; Zhen *et al.* 2012; Conte *et al.* 2015; Meyer *et al.* 2018), most tend to focus on traits controlled by only a handful of genes. Fewer still connect what occurred in nature to results obtained in the laboratory. One outstanding question would be whether parallelism occur only when relatively fewer beneficial alleles segregate in the starting populations (Chan *et al.* 2012; Hillis *et al.* 2020), and/or when such alleles respond particularly strongly to selection (Orr 2005; Castro *et al.* 2019). We therefore do not know if the same rules of parallelism or convergence hold for more complex traits like body size and weight (but see Chan *et al.* 2012; Castro *et al.* 2019).

Island populations of mice represent an outstanding opportunity to study parallel evolution: the house mouse is exceedingly successful in colonizing diverse habitats, including numerous remote islands, many at high latitudes. Following colonization, they have often evolved to large body size (Foster 1964; Lomolino 1985). Such events, repeated over and over again, represent replicated natural experiments. There are many parallels to changes in body size and weight in laboratory mice, which have been the subject of many classical quantitative genetics, developmental genetics and physiology studies (MacArthur 1944; Cheverud 1996; Bünger *et al.* 2001; Renne *et al.* 2006; Chan *et al.* 2012). Together, these resources present a unique opportunity to study the genetic basis of evolutionary change in nature, and to make connections to results obtained among laboratory strains.

We chose to study the natural mouse population on the Faroe Islands, because of their body size and as a representative of the mice in the North Atlantic. The Faroe Islands are an island group of 18 major islands in the North Atlantic, six of which are inhabited by house mice. Since their discovery more than a century ago, the Faroese house mice have attracted considerable interest, on account of their large body size and distinctive morphology (Eagle Clarke 1904). So remarkable were the Faroese mice that they have variously been classified as the new subspecies *faeroensis*, or even *mykinessiensis*, from a single island Mykines of only 10 km^2^ in area (Eagle Clarke 1904; Degerbøl 1942; Berry *et al.* 1978a).

To date, the population on Gough Island in the mid/South Atlantic represents the most extensively studied thus far in the context of body size/weight increase in house mouse (Gray *et al.* 2015; Parmenter *et al.* 2016). Whereas the continental source population of the mice on the remote Gough Island remains unclear (Gray *et al.* 2014), the Faroese mice show close links to other Northern Atlantic populations in morphology and genetics (Berry *et al.* 1978a; Jones *et al.* 2011; 2012). Specifically for genes controlling body size and weight, we have previously shown evidence from mandibles of wild-caught mice indicating their large size, as well as detecting evidence of selective sweeps in the Faroese mice at two loci found to be involved in body weight changes of selected laboratory mice (Chan *et al.* 2012). This makes the Faroese mice a compelling example of the island effect for a large-scale genetic mapping study.

## Materials and methods

### Field sampling

House mouse (*Mus musculus spp.*) was sampled with live traps (DeuFa, Neuburg, Germany) on Mykines over successive nights in October 2009. In total, 20 mice were caught and introduced into the Mouse House at the Max Planck Institute for Evolutionary Biology in Plön, Germany. Sampling locations and numbers are shown in Supplementary Table S1. Due to the small size of Mykines, all sampling locations were less than 1 km apart and were treated as a single locality. The mice were then paired, where possible, within sampling sites to establish the MYK strain (Supplementary Table S1).

### Animal care and use

All experimental procedures described in this study have been approved by the local competent authorities: the Faroese Food and Veterinary Agency and Ministry for Agriculture, Environment and Rural Area, Schleswig-Holstein, Germany (Permit number 97-8/07).

#### Genetic cross and phenotyping

Mice from MYK and SM/J strains bred in our facility under common-garden conditions were crossed with each other in reciprocal directions to establish 12 F1 families, which in turn generated 841 F2 mice (Supplementary Tables S1 and S2). Mice were weaned at 4 weeks of age. Each F2 mouse was housed singly for 16 weeks, and were weighed biweekly for a total of 7 time-points. At weeks 8 and 16, body and tail length measurements were obtained under anesthesia (week 8) or immediately following sacrifice (week 16). In addition, blood plasma was prepared following the protocol by Yuan *et al.* 2009. Briefly, each animal scheduled for sacrifice was fasted for 4 h (from 9am), and was sacrificed between 2pm and 5pm. Immediately upon sacrifice, the mice were dissected, and blood drawn from the heart was spun down with a table-top centrifuge. In addition, liver was dissected and weighed. Ear clips were taken for the purpose of DNA extraction.

#### Growth curves

In addition to biweekly weight measurements, we also estimated parameters related to growth in each mouse using the grofit package in R (Kahm *et al.* 2010), which estimated for each mouse the growth rate μ and asymptote A parameters.

#### Growth hormone measurements

Plasma level of growth hormone *Insulin-like growth factor 1* (*Igf1)*, *IGF* binding protein 2 and 3 (*Igfbp2* and *Igfbp3*) were determined using enzyme-linked immunosorbent assay (ELISA) kits (ALPCO, Salem, NH, USA), according to manufacturer’s instructions. Colorimetric reactions were quantified by measuring absorbance at 450 nm using a Tecan Infinite M200 PRO (Tecan AG, Schwerte, Germany) microplate reader equipped with MAGELLAN 7.0 software. Samples were randomized in their positions, and each sample was measured in duplicates. A standard curve was included in every sample plate and was used to estimate concentration. A subset of samples was measured an additional time to determine repeatability. Repeatability for *Igf1*, *Igfbp2* and *Igfbp3* was determined to be 0.95, 0.94 and 0.78, respectively (*Igf1*, *n* = 43; *Igfbp2*, *n* = 44; *Igfbp3*, *n* = 48, estimated using the rpt.aov function in rptR (Stoffel *et al.* 2017).

### Data availability

All raw data and code are deposited at the following repository: https://github.com/evolgenomics/FaroeQTL. Raw sequence reads have been deposited at NCBI under BioProject accession PRJNA684612. 

Supplementary material is available at figshare DOI: https://doi.org/10.25387/g3.13369277

#### Reference genome assembly

All coordinates in the mouse genome refer to *Mus musculus* reference mm10, which is derived from GRCm38.

#### Restriction sites-associated DNA sequencing

Given the large size of the mouse genome and the coverage required to confidently call genotypes, we chose to use Restriction sites-associated DNA sequencing (RADseq) to concentrate the sequencing around rare restriction cut-sites. RADseq was performed according to Poland *et al.* 2012, and specifically with the same reagents as used in Witte *et al.* 2015, with the following modifications. Instead of *PstI* (a 6-cutter with the recognition motif C, TGCA’G), *SbfI-*HF (New England Biolabs GmbH), which recognizes the 8-nt motif CC, TGCA’GG but shares the same TGCA 3’ overhang, was used together with *MseI*, in order to further enrich the sequencing library for a smaller subset of sites. DNA from the F2 panel of 841 mice were extracted from ear clips. The DNA from each mouse was double digested with *SbfI*-HF and *MseI* and ligated to the adapters. The resulting library was subjected to size selection (400–600 bp) using gel electrophoresis. The library was normalized to the same DNA concentration. Sets of 94 libraries were pooled together, amplified by thermocycling using universal primers and sequenced by a HiSeq 2000 (Illumina Inc., San Diego, CA, USA) at the Genome Core Facility at the MPI Tübingen Campus. The overall sequencing output was inspected and about 10% of the samples re-run to ensure sufficient sequence coverage for genotype calling.

#### Sequence demultiplexing and genotyping pipeline:

The F2 panel was sequenced across a total of 11 HiSeq2000 lanes, including re-runs. In each lane, the sequencing data was pre-processed and demultiplexed using the package Short Read (SHORE; Ossowski *et al.* 2008). Briefly, fastq files from each lane were demultiplexed into each well via a set of in-line barcodes (5–10 nt) in Read1 with the parameter –barcode-mismatches = 1. In addition to the F2 samples, the two grandparental samples were separately whole-genome shotgun sequenced to approximately 15x coverage by a HiSeq2000 (Illumina) at the Cologne Center for Genomics. Sequence data were pre-processed using a pipeline consisting of data clean-up, mapping, base-calling and analysis based upon fastQC v0.10.1 (2016) ; trimmomatic v0.33 (Bolger *et al.* 2014); bwa v0.7.10-r789 (Li and Durbin 2010); GATK v3.4-0-gf196186 modules BQSR, MarkDuplicates, IndelRealignment (McKenna *et al.* 2010; DePristo *et al.* 2011). Genotype calls were made using a pipeline consisting of samtools mpileup and bcftools call module under the multiallelic mode. The raw genotype calls were filtered using the parameters TYPE=“snp” && N_SAMPLES > 100 && MAF > 0.25 and only informative positions from the two parents were retained. Using custom Perl scripts, we used the two parental lines to polarize the genotypes and averaged the frequency calls over sliding windows of 250 kbp by 50 kbp steps. These form the genotype datasets we used for the subsequent linkage mapping step.

#### Linkage mapping

Linkage mapping was performed in R using the packages R/qtl (Broman *et al.* 2003) and R/qtlRel (Cheng *et al.* 2010). Gentoype data was coded as “M” for Mykines, “H” for heterozygous and “S” for SM/J. Due to the use of the Faroe mouse parental line, we rebuild a genetic map from this dataset using the Kosambi map function. The resulting map has a total length of 2379 cM (chromosome span: 65–200 cM). In total, 22 phenotypes were retained for this analysis, consisting of weight, length and plasma protein measurements, as well as a set of covariates such as sex, family history, age and cross directions. We applied Box–Cox transformation to numeric datasets, resulting in z-standardized, mean-centered phenotypes with improved normality. Genetic mapping was performed on the transformed dataset. We also obtained major axes of variation through principal component analysis. For each trait, we applied corrections for family, sex and cross directions following a backward model selection procedure in which we simplify from a full additive and interactive set of covariates to the minimal set based on the Aikake Information Criterion (AIC). Genome-wide significance thresholds were determined from 1000 permutations. For QTL mapping with relatedness correction under QTLrel, relatedness was estimated from marker genotypes at non-focal chromosomes and was fitted as a random effect. Following QTL detection, the effect sizes were estimated by fitting a QTL model against the original, untransformed phenotypes.

### Meta-analysis

The analysis across different mapping panel was performed used QTLrel (Cheng *et al.* 2010), following closely the procedure described in Parker *et al.* 2014. Briefly, phenotype and genotype data from the Cheverud and Palmer labs, as well as from Stylianou *et al.* 2006 were obtained (Cheverud 1996; Stylianou *et al.* 2006; Norgard *et al.* 2008; Parker *et al.* 2014). The genotype data was coded as the number of SM/J allele in each panel. Then QTL mapping with relatedness correction was performed, using marker-calculated measures of relatedness. Since our main focus here is the broader comparison of mapping results, we did not attempt extensive bootstrapping analyses to determine the significant threshold in the combined analysis.

## Results

To enable direct comparisons between laboratory and wild mice in their genetic basis of body size and weight, we organized a field expedition to collect live mice from the Faroe Islands. We focused on Mykines (“MYK”), the westernmost major Faroese island, because its mouse population was consistently the largest and most distinctive in the Faroese archipelago (mean adult weight 33.5 g, *N* = 9 in 1999, range: 27.4–39.9 g *vs* 15.3 g from Tórshavn, capital and active port receiving Danish shipments, thus likely of continental origin, *N* = 7) (Eagle Clarke 1904; Degerbøl 1942; Berry *et al.* 1978a; Jones *et al.* 2011). For the purposes of line crosses, the MYK population also were most inbred (Jones *et al.* 2011), making it likely that the sampled individuals would be representative of the population at large. Following a generation of breeding under common conditions, we set up crosses with the small SM/J strain to determine firstly the genetic basis of body weight and length variation using a quantitative trait locus (QTL) mapping approach, and additionally to assess the extent of QTL sharing with other examples of bodyweight evolution, principally by a combined QTL meta-analysis using previously published QTL studies involving the SM/J strain, mostly against the large LG/J line (Cheverud 1996; Stylianou *et al.* 2006; Parker *et al.* 2011).

**Figure 1:**
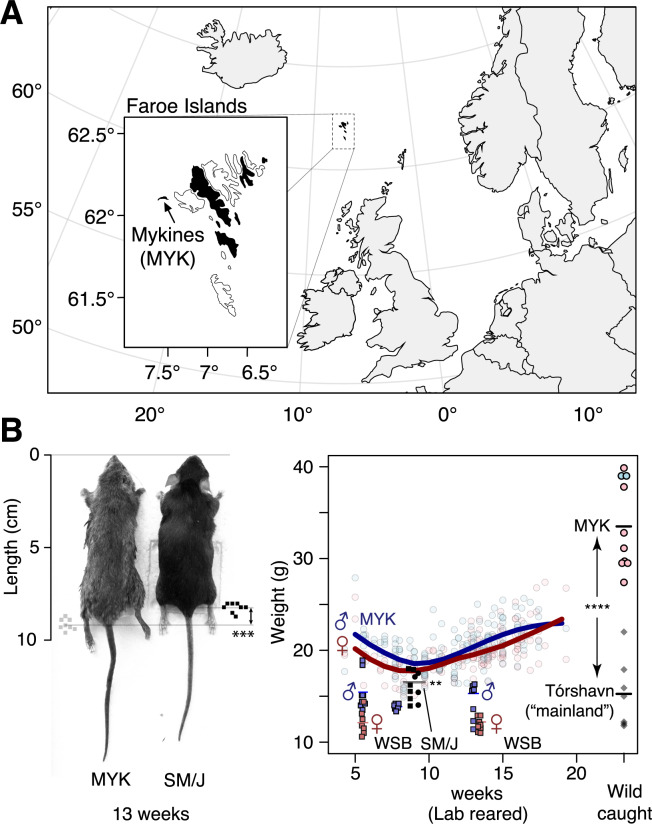
The large-bodied Faroese house mouse. (A) The Faroe Islands are an island group of 18 major islands in the North Atlantic (inset), six of which (marked in black) are home to wild house mice (*Mus musculus*, mainly of *domesticus* subspecies, historically *faeroensis*; black) (Berry *et al.* 1978a). Mykines (MYK), the westernmost island measuring 10 km^2^, is home to a distinctive mouse population (“*mykinessiensis*”). (B) These mice are among the largest wild mice in the Faroe Islands and the world. A genetic cross was set up between the MYK mice (grey) and the laboratory strain SM/J (black) to investigate firstly the genetic basis of length (left) and weight variation (right) in the Faroese mice, and secondly the extent of sharing in the genetic loci underlying body weight changes between laboratory and wild mice. Wild-caught MYK mice weigh between 29 and 40 g (right, circles; blue: male; pink: female) and they are distinctly larger than mice sampled from Tórshavn (diamonds), likely representatives of continental origin. In the laboratory colonies, MYK mice weigh less, but still heavier than other representative strains, *e.g.*, WSB at 6 and 13 weeks of age, or SM/J at 8 weeks of age, implying both environmental and genetic contributions to increased body weight. **P < 0.004; ****P < 1 × 10^−7^. See main text for details on statistical comparisons.

Overall, the MYK lab-bred male mice were significantly heavier than SM/J mice both at 8 and 13 weeks of age (Figure 1B; Weight at 8 weeks, males: 19.5 *vs* 16.0 g: *N* = 12, 10; *t *=* *3.18, df = 12, *P *<* *0.004; 13 weeks, males: 22.5 *vs* 18.9 g, *N* = 17, 8; *t = *3.1, df = 6, *P *<* *0.004). Females showed a smaller difference along similar trends (Figure 1B; weight at 8 weeks in females: 17.8 *vs* 16 g, *N* = 13, 10: *t *=* *1.6, df = 6, *P *=* *0.08; 13 weeks females: 20.5 *vs* 18.1 g, *N* = 29, 8; *t *=* *1.5, df = 11, *P *=* *0.08). Male MYK were also significantly longer than SM/J at 13 weeks (9.2 *vs* 8.2 cm; Student’s *t*-test: *N* = 24, 8; *t *=* *6.7, df = 13.6, *P *<* *5.8 × 10^−6^). Interestingly, we observed a drop in laboratory-bred MYK body weight compared to wild-caught individuals, a finding also reported previously in laboratory populations of Gough Island mice (Gray *et al.* 2015). This suggests that the environmental conditions on the islands add significantly to the genetic contributions of increased body weight.

**Figure 2:**
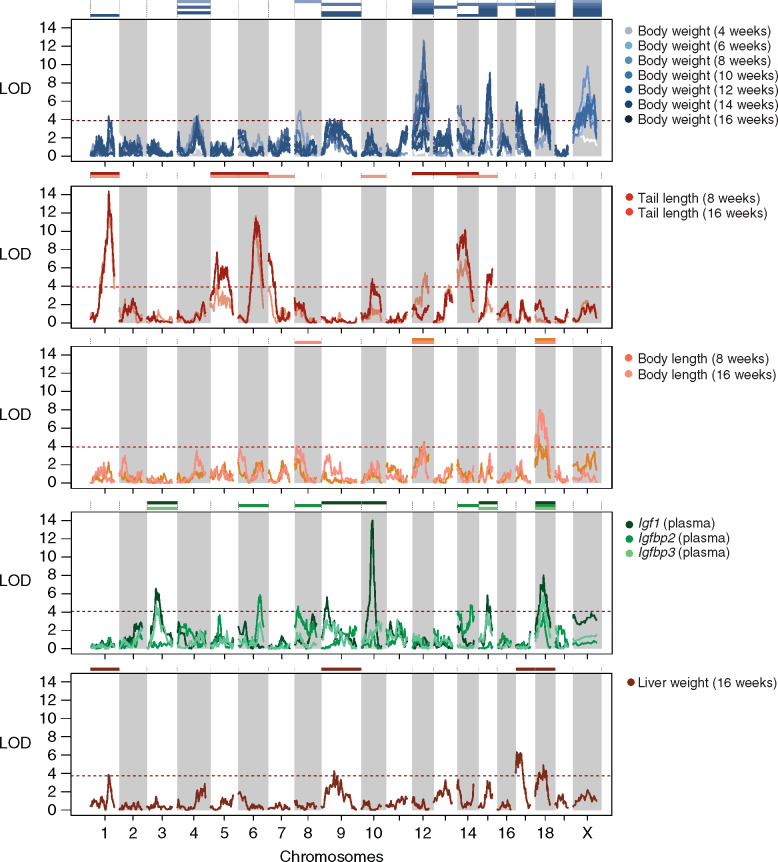
Genetic associations for the measured phenotypic traits in five groups. Linkage mapping was performed to detect loci affecting body weight (blue); tail lengths (red); body lengths (orange); growth hormone levels (green) and liver weight (brown; *x*-axis: chromosomes; *y*-axis: log of the odds statistical support for a quantitative trait locus or QTL affecting a trait). In total, we detected 82 QTLs at the genome-wide significant threshold of ∼3.9 (red dotted line) on 17 out of 20 chromosomes (QTL: colored bars over the chromosome).

The mapping pedigree was started with first-generation, lab-reared MYK mice derived from the most successful laboratory cross among wild-derived mice caught at the same trapping sites (Supplementary Table S1). This had the effect of further insulating us from environmental effects and removing more of the remaining variation within the MYK strain, which could improve the power to detect differences against the SM/J alleles. From among these full-sib offspring, we generated crosses between 5 MYK female × SM/J males and 2 SM/J × MYK reciprocal pairs from which 12 F1 full-sib families were derived to generate a total of 841 F2 mice (Supplementary Figure S1). For each F2 mouse, we collected a total of 17 traits in 5 groups (body weight, total length and tail length a different ages, *Igf* pathway proteins and liver weight at 16 weeks of age; Tables 1 and 2, and Figure 2). Males were on average heavier than females, weighing 15.9–22.4 g from week 4 to 16 *vs* 14.7–19 g in females. We observed a slight, but significant parent-of-origin effect, but only in females: female mice having an MYK paternal grandmother tended to be born lighter, but gained more weight through to adulthood (Supplementary Figure S2; *N* = 4242 observations in 606 individuals, repeated-measures ANOVA with time and cross direction; *P *<* *0.89 in males and 0.0003 in females). In an F2 intercross design, all females but not males consistently carry the paternal grandmother X chromosome, this effect was consistent with the MYK X chromosome being associated with lighter birth weights but greater growth rate over 16 weeks.

**Table 1:** QTL locations and effect sizes

			Peak			Conf. int.				
Trait (unit)	QTL name	Chr	Mbp	cM	LOD	Mbp	Genes	%VE	*a*	*d*
*Igf1* plasma level (ng/ml)	*igf1q3*	3	73.5	43	6.59	67–117	557	4.9	−34.9	3.4
	*igf1q9*	9	33.5	27	5.63	24–41	193	4.2	−31.6	−6.8
	*igf1q10*	10	84.5	55	14.00	74–88	297	10.1	56.1	−7.3
	*igf1q15*	15	73.5	43	5.83	69–92	352	4.3	−26.1	7.3
	*igf1q18*	18	58	40	8.00	42–61	104	5.9	−43.9	2.4
*Igfbp2* plasma level (ng/ml)	*igfbp2q6*	6	134	107	5.88	124–138	206	4.4	40.5	−21.3
	*igfbp2q8*	8	19	17	4.64	17–20	22	3.5	−35.0	8.2
	*igfbp2q14*	14	99	65	4.78	17–113	725	3.6	37.2	−13.8
*Igfbp3* plasma level (ng/ml)	*Igfbp3q3*	3	76	45	4.86	74–110	513	3.6	−12.9	7.0
	*Igfbp3q18*	18	55.5	40	5.80	42–61	104	4.3	−17.2	4.2
Body weight (6 weeks, g)	*bw6wq4*	4	110	103	4.50	86–111	183	3.5	0.46	−0.29
	*bw6wq8*	8	26.5	26	4.97	17–32	146	3.8	0.55	−0.20
	*bw6wq12*	12	92	56	6.99	30–95	356	5.4	0.74	−0.24
	*bw6wqX*	X	99	69	6.18	74–142	339	2.2	0.10	0.05
Body weight (8 weeks, g)	*bw8wq9*	9	43	40	4.03	42–44	11	3.2	−0.41	0.08
	*bw8wq12*	12	90	55	12.62	90–94	7	9.8	0.91	−0.07
	*bw8wq14*	14	11.5	2	5.52	6–32	194	4.4	0.64	−0.21
	*bw8wq15*	15	74	44	6.05	68–92	352	4.8	−0.49	0.19
	*bw8wq16*	16	27	21	3.90	27–34	63	3.1	−0.56	−0.41
	*bw8wq17*	17	47.5	27	4.00	46–48	40	3.2	0.47	−0.27
	*bw8wq18*	18	38.5	22	4.71	30–68	326	3.8	−0.58	0.17
	*bw8wqX*	X	100	71	9.89	74–101	141	5.6	0.87	0.39
Body weight (10 weeks, g)	*bw10wq4*	4	103.5	99	4.23	86–110	183	3.3	0.70	0.15
	*bw10wq12*	12	90.5	55	11.92	90–94	7	9.1	1.04	−0.09
	*bw10wq13*	13	103	75	4.07	103–108	22	3.2	0.60	0.42
	*bw10wq15*	15	74	44	4.48	68–92	352	3.5	−0.51	0.15
	*bw10wq18*	18	55.5	40	5.43	31–68	325	4.2	−0.69	0.41
	*bw10wqX*	X	100	71	5.38	54–158	546	3.1	0.93	0.54
Body weight (12 weeks, g)	*bw12wq12*	12	94.5	58	8.37	84–100	97	6.3	0.93	0.19
	*bw12wq15*	15	90.5	55	7.61	72–92	350	5.7	−0.77	0.14
	*bw12wq17*	17	7.5	5	4.76	4–48	800	3.6	0.62	0.27
	*bw12wq18*	18	59.5	42	4.52	31–68	325	3.4	−0.63	0.17
	*bw12wqX*	X	100	71	5.10	10–163	802	2.8	0.91	0.50
Body weight (14 weeks, g)	*bw14wq4*	4	100	95	4.37	100–102	20	3.4	0.60	−0.35
	*bw14wq9*	9	57	63	4.05	57–58	25	3.2	−0.48	0.55
	*bw14wq12*	12	92	56	5.26	87–94	43	4.1	0.81	−0.14
	*bw14wq15*	15	90.5	55	9.13	74–92	338	7.0	−0.83	0.30
	*bw14wq17*	17	46.5	27	5.05	4–50	822	3.9	0.70	−0.45
	*bw14wq18*	18	58	40	7.70	30–61	272	5.9	−0.88	0.26
	*bw14wqX*	X	100	71	6.76	74–163	458	3.4	1.14	0.68
Body weight (16 weeks, g)	*bw16wq1*	1	163	89	4.38	163–167	40	3.3	0.49	0.30
	*bw16wq9*	9	44	40	4.06	42–44	11	3.0	−0.62	−0.06
	*bw16wq14*	14	120	80	4.18	119–121	6	3.1	0.40	−0.43
	*bw16wq15*	15	74	44	7.85	68–92	352	5.8	−0.74	0.03
	*bw16wq17*	17	7.5	5	5.92	1–41	709	4.4	0.68	0.08
	*bw16wq18*	18	41.5	25	7.93	31–61	271	5.8	−0.89	−0.02
	*bw16wqX*	X	100	71	5.71	56–161	553	2.6	1.09	0.69
Liver weight (16 weeks, g)	*Livwq9*	9	57	63	4.26	57–58	25	3.3	−0.02	0.03
	*Livwq17*	17	8	5	6.32	4–55	834	4.9	0.05	0.01
	*Livwq18*	18	58	40	4.90	31–68	325	3.8	−0.05	−0.02
Total length (8 weeks, cm)	*Ltot8wq1*	1	163.5	90	8.56	154–167	105	7.0	0.24	0.09
	*Ltot8wq5*	5	89	59	3.99	88–89	29	3.3	−0.14	−0.07
	*Ltot8wq6*	6	101.5	88	8.17	96–132	315	6.7	0.23	−0.06
	*Ltot8wq12*	12	94.5	58	6.44	35–108	421	5.3	0.23	−0.08
	*Ltot8wq14*	14	11.5	2	5.59	6–30	145	4.6	0.24	−0.10
	*Ltot8wqX*	X	101.5	71	4.05	98–159	329	2.0	0.89	0.43
Total length (16 weeks, cm)	*Ltot16wq1*	1	163	89	6.51	128–180	456	4.8	0.20	0.02
	*Ltot16wq5*	5	44	32	5.46	30–110	534	4.1	−0.16	−0.20
	*Ltot16wq6*	6	131.5	104	7.53	91–137	408	5.6	0.21	−0.12
	*Ltot16wq7*	7	23.5	3	4.21	22–25	78	3.2	−0.15	0.02
	*Ltot16wq10*	10	85.5	55	4.15	14–32	446	3.1	0.16	0.03
	*Ltot16wq14*	14	8	0	6.12	74–118	586	4.5	0.23	−0.06
	*Ltot16wq15*	15	92	55	5.24	87–94	530	3.9	−0.18	0.01
	*Ltot16wq18*	18	44	26	6.45	101–103	272	4.8	−0.21	−0.01
Tail length (8 weeks, cm)	*TailL8wq1*	1	167	90	11.88	156–182	286	9.4	0.17	0.02
	*TailL8wq5*	5	44	32	4.16	44–46	6	3.4	−0.08	−0.05
	*TailL8wq6*	6	101.5	88	11.67	87–108	122	9.3	0.15	−0.01
	*TailL8wq12*	12	97	64	5.44	84–108	200	4.4	0.13	−0.04
	*TailL8wq13*	13	103	75	4.10	103–103	1	3.4	0.11	0.01
	*TailL8wq14*	14	61.5	41	6.88	6–82	749	5.6	0.12	0.02
Tail length (16 weeks, cm)	*TailL16wq1*	1	167	90	14.35	162–170	63	10.5	0.18	0.01
	*TailL16wq5*	5	44	32	7.71	38–128	642	5.8	−0.12	−0.10
	*TailL16wq6*	6	101	88	11.43	87–134	437	8.4	0.15	0.01
	*TailL16wq7*	7	23	3	7.56	17–34	463	5.7	−0.11	0.01
	*TailL16wq10*	10	85.5	55	4.80	74–100	364	3.6	0.10	0.00
	*TailL16wq14*	14	58	39	10.15	6–64	615	7.5	0.14	0.03
	*TailL16wq15*	15	101	67	5.89	72–103	542	4.4	−0.07	−0.06
Body length (8 weeks, cm)	*BodyL8wq12*	12	94.5	58	4.50	62–94	242	3.8	0.12	−0.03
	*BodyL8wq18*	18	31.5	18	5.12	31–68	325	4.3	−0.11	−0.03
Body length (16 weeks, cm)	*BodyL16wq8*	8	14.5	10	4.07	14–16	7	3.1	0.10	−0.05
	*BodyL16wq12*	12	92	56	4.26	87–94	43	3.2	0.11	−0.03
	*BodyL16wq18*	18	38.5	22	8.03	28–61	272	6.0	−0.13	0.04
PC1	*PC1q1*	1	163.5	90	5.35	162–170	66	4.0	−0.54	−0.32
	*PC1q4*	4	100	95	4.44	100–102	21	3.3	−0.67	0.21
	*PC1q6*	6	131.5	104	4.24	130–132	6	3.2	−−0.53	0.42
	*PC1q12*	12	92	56	7.97	85–94	70	5.9	−0.94	0.23
	*PC1q14*	14	11.5	2	6.04	6–32	194	4.5	−0.79	0.27
	*PC1q15*	15	74	44	7.42	72–92	350	5.5	0.65	−0.15
	*PC1q17*	17	46.5	27	3.92	46–48	40	2.9	−0.54	0.45
	*PC1q18*	18	41.5	25	8.16	31–68	325	6.0	0.85	−−−0.03
	*PC1qX*	X	100	71	6.45	74–160	443	3.7	−1.03	−0.59
PC2	*PC2q1*	1	104	60	7.54	90–186	644	5.6	0.38	0.06
	*PC2q3*	3	73.5	43	4.27	74–107	458	3.2	0.24	−0.09
	*PC2q6*	6	100	87	5.12	87–108	122	3.8	0.29	−0.04
	*PC2q9*	9	57	63	4.60	53–57	51	3.4	0.37	−0.26
	*PC2q18*	18	58.5	42	3.89	52–61	49	2.9	0.25	−0.05
PC3	*PC3q1*	1	137	77	3.93	134–138	57	2.9	−0.23	0.08
	*PC3q5*	5	131.5	88	4.58	30–136	844	3.4	0.16	0.27
	*PC3q6*	6	124	99	6.99	75–138	567	5.2	−0.33	−−0.03
PC5	*PC5q8*	8	23	19	6.50	17–46	207	4.8	−0.17	−0.11
	*PC5q13*	13	17	8	4.06	17–17	1	3.0	0.20	−0.04
PC6	*PC6q2*	2	167	96	5.91	162–180	214	4.4	0.19	−−0.17
	*PC6q8*	8	15	10	5.75	10–28	193	4.3	0.22	−0.05
	*PC6q9*	9	108.5	158	4.32	108–122	161	3.2	0.24	0.14
	*PC6q14*	14	98	65	5.14	56–110	247	3.8	−0.19	0.05
PC7	*PC7q11*	11	92.5	70	4.48	66–96	534	3.4	−−0.15	0.13
PC8	*PC8q12*	12	72	44	5.50	53–100	300	4.1	0.17	−0.05
PC10	*PC10q10*	10	87.5	56	7.99	74–91	321	5.9	0.18	−0.01
PC12	*PC12q10*	10	83.5	53	19.90	82–86	30	14.0	0.24	−0.05
PC13	*PC13q10*	10	86.5	56	7.88	68–91	338	5.8	−0.15	0.03
PC18	*PC18qX*	X	75	52	5.92	5–170	919	4.4	0.14	0.14

Chr, Chromosome of the QTL; Mbp, Physical position of the peak marker for the QTL, given in 0.5 Mbp windows; cM, Genetic position of the peak marker, in centimorgan; Conf. int.; 2-LOD confidence interval; LOD; Log of the odds, indicating statistical support for a QTL at a given location; %VE, Percentage of variance explained by QTL; *a*, Additive coefficient, showing the effect per copy of MYK allele; *d*, Dominance coefficient.

To perform quantitative trait locus (QTL) mapping to find associations between the genotype and phenotype data, we sequenced the parental lines at 15-fold coverage and constructed a genetic map using offspring genotypes (*N* = 606) from a two-enzyme version of restriction-site associated DNA sequencing (RAD-seq; Poland *et al.* 2012; Witte *et al.* 2015).

We approached the genetic mapping in three steps. First, we mapped each measured trait singly. Following Parker *et al.* (2014) we also correct for genetic relatedness, even among F2 mice, in our mapping analysis for improved power to detect QTLs. Then we focused on the underlying structure of the data by either fitting growth curves over the entire growth series in each individual, or by extracting the major axes of variation in the dataset via principal components analysis. Finally, we performed a composite joint mapping by integrating data from additional QTL datasets involving SM/J, in order to examine the extent of QTL sharing between laboratory mice and MYK mice.

Overall, our mapping revealed a strong genetic basis for trait variation in this cross. For all but one of the 17 measured traits, we found 2 to 8 QTLs that together explained on average 24% of the variance in a trait (Table 1; median: 5 QTLs per trait, explaining on average 4.9%; combined, the QTLs explain 8–45.9% of variance in a given trait).

Among the strongest QTL is a locus on Chr10 (84.5 Mbp), *igf1q10*, that controls the blood plasma level of the growth hormone *Insulin-like growth factor 1* (*Igf1*, LOD: 14.0, 10% variance explained). This QTL overlaps with the *Igf1* gene itself (Chr10: 87.9 Mbp) and a previously reported QTL *Igf1q4* involving SM/J and another laboratory strain MRL/MpJ (Leduc *et al.* 2010) that implicates a 5’UTR C/A variant rs29342496. Here, unlike the SM/J C variant, MYK carries an A at rs29342496, which we estimated to increase IGF-1 level by 56 ng/ml per allele (*vs* an average of 391 ng/ml among F2 mice). This is largely consistent with the contrast seen in crosses between laboratory strains (Rosen *et al.* 2000; Leduc *et al.* 2010). Like those previous studies, we observe no protein-coding changes at *Igf1* between MYK and SM/J, ruling out coding changes that affect the protein's activity, stability, or degradation. Taken together, our data suggest that SM/J likely carries a regulatory variant affecting the circulating IGF-1 protein level.

The strongest QTL in this dataset, *TailL16wq1*, is located on Chr1 (167 Mbp) and controls variation in tail length (16 weeks, LOD: 14.4, 10.5% variance explained). This locus—or other tightly linked ones—also controls tail length at 8 weeks, and body weight at 16 weeks. Unlike *igf1q10* on Chr10 for IGF-1 plasma level, this is a morphological trait and not a gene-specific one. Close examinations of the confidence interval (Chr1:161.5–169.5 Mbp) revealed 63 protein-coding genes, including the genes *Suco* and *Lmx1a*, whose knockout phenotypes show bone ossification and tail phenotypes, as well as multiple classical spontaneous short tail mutants for *Lmx1a* (Wahlsten *et al.* 1983; Sohaskey *et al.* 2010). Overall, our data suggest that the tail of a mouse may be governed by a smaller set of loci, because for this trait we could detect three major QTLs with >10 LOD support, with 9 total QTLs (6 shared) between the 8- and the 16-week measurements. In fact, at week 16 of age, the seven QTLs could together explain nearly half of the variance.

We next turn to body weight measurements. As expected, biweekly weight measurements are highly correlated (mean r = 0.69, range: 0.39–0.9, 7 time points, *N* = 606; Supplementary Table S3). We thus observe that many single trait QTLs overlap, with genome-wide significant QTLs on Chr12, 15, 17, 18, and X at multiple time-points. Except for the lack of genome-wide significant QTLs at week 4, there is not a clear trend between the number of QTLs (range: 3–8) and the timing of the measurement. Across all growth QTLs, the *bw8wq12* at Chr12: 90 Mbp (significant for 6–14 weeks, 2-LOD interval: 89.5–94.5 Mbp) has the highest LOD, peaking at 12.6 in week 8. This QTL covers only 7 genes, including *Thyroid-stimulating hormone receptor* (*Tshr*), a compelling candidate that plays a central role in metabolism and growth regulation principally through the pituitary–hypothalamus axis (Postiglione *et al.* 2002). At *Tshr* we also did not find any non-synonymous mutations. Here, the decreasing effect of the QTL as the mouse aged drew us to an observation for the spontaneous *Tshr^hyt-2J^* allele, in which the growth retardation effect was most obvious during the first 8 weeks, and its fertility defect can be greatly mitigated if weaning was delayed till week 12–18 (Dionne *et al.* 2014).

The remaining QTLs tend to have larger QTL intervals (median 2-LOD interval span: 32 Mbp) and/or contain many more genes (median: 272). Thus we refrain from proposing candidate genes in these intervals.

We then attempted mapping and interpreting broader growth curves and major variation modes. For instance, we can summarize the growth series by fitting Gompertz growth functions (Gompertz 1825), focusing on two parameters: the maximal growth rate *µ* and the final weight or asymptote *A*. The obvious advantage of fitting growth curves is to capture the growth dynamics in an individual animal. However, the assumptions and errors associated with growth curves fitting may also obscure potentially important observations, such as large variations in weaning weight at 4 weeks or drops in body weight over time. Here, we were able to estimate growth parameters for *µ* in 229 and for *A* in 216 individuals, and detected a single suggestive QTL for the asymptote weight *A* at Chr18: 58 Mbp that overlaps with that of the bodyweight QTLs from weeks 10–16. Notably, this QTL also overlaps *igf1q18* and *igfbp3q18*, underscoring the functional involvement of the *Igf1* pathway in post-pubertal growth (Styne 2003; Stratikopoulos *et al.* 2008; Courtland *et al.* 2011).

In 17 out of 26 distinct QTLs described above (from 82 overlapping QTLs), the MYK allele is associated with increased weight and length (65.4%, binomial sign test, *P* ∼ 0.08, *h *>* *0; or 50 out of 82 raw QTL without collapsing, 61.0%). The effect sizes of these 50 MYK-increasing QTLs are also greater (0.98 *vs* 0.14; Supplementary Figure S3). Both lines of evidence were consistent with MYK being the heavier parental strain.

**Figure 3:**
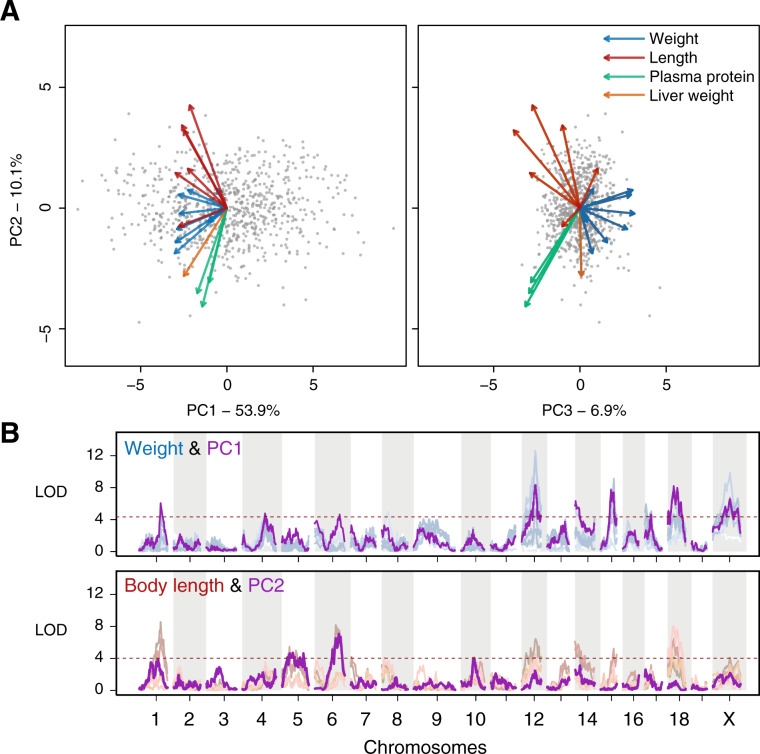
Major modes of variation in body weight and length summarize and recapitulate trait types. (A) Individual measurements were resolved into independent modes of variation using a principal components analysis (PCA). There is a strong correlation across all traits, as indicated by the shared direction of each of the trait vectors (each dot is an F2 individual. Trait vectors are colored according to trait types; see also Supplementary Table S3). (B) Mapping of the PCs (purple) show LOD profiles that largely track with the individual trait types: PC1 with weight and PC2 with body length.

Another way to summarize QTLs is to extract the major axes of variation within the trait data using principal component analysis (PCA), and map each as a composite trait. In effect, we use PC traits as a way to isolate and map mutually independent growth modes. Visualization of the top two principal components (PC1 and 2, accounting for 54% and 10%, respectively) shows that all the measured traits are strongly correlated with each other (Supplementary Table S3), with their eigenvectors all loading negatively on PC1 (Figure 3A). Beyond the first level, the different types of traits quickly split into independent directions, such that PCs 2 and 3 effectively summarizes the contrast between length, weight, liver weight and plasma protein levels.

**Figure 4:**
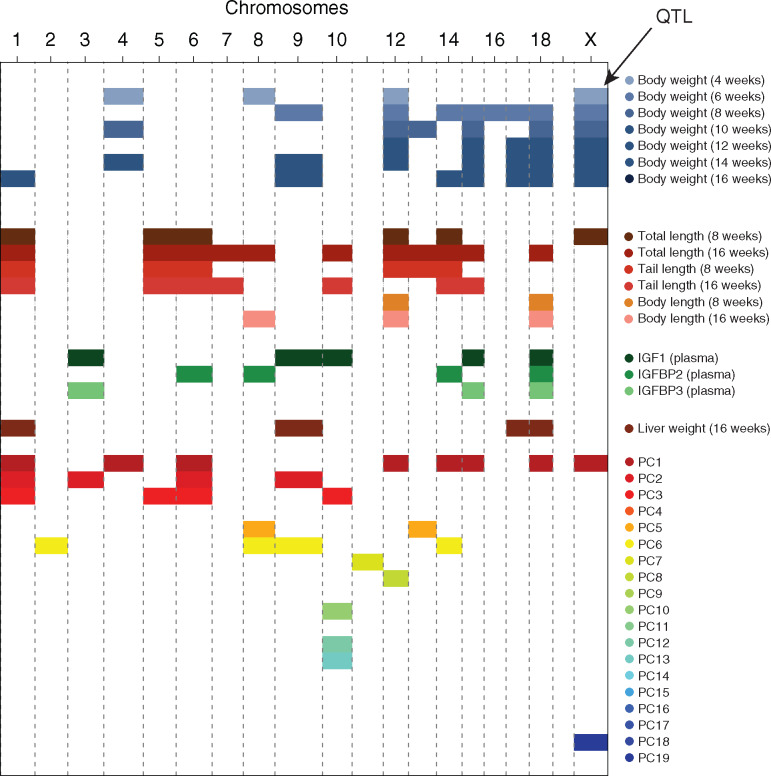
Chromosomal distribution of trait associations. A summary representation of all of the significant QTLs recovered in the current study shows that while there is a strong auto-correlation and sharing within a given trait type (weight, size, *etc*.), the set of chromosomes carrying genome-wide significant QTLs appear to be largely distinct from one type of traits to another. This also results in the absence of a single or few “super-gene” clusters that would account for much of the observed variation in the traits.

Mapping of these PCs both recapitulates the major QTLs, but also revealed additional loci not discovered in single-trait analysis, *e.g.*, *PC2q1* at Chr1: 104 Mbp and *PC3q5* at Chr5: 132 Mbp (Table 1). More broadly, PC1 mostly summarizes the bodyweight variation (Figures 3B and 4). In the case of the 9 genome-wide significant PC1 QTLs (and two additional suggestive QTLs), they show a classical oligogenic architecture associated with body weight and growth.

A global comparison of the chromosomes carrying QTLs across the different trait types is shown in Figure 4. All traits are controlled by several loci scattered across the genome and tend not to cluster into single major-effect chromosomes.

We next asked if there was evidence of QTL sharing in examples of parallel body weight changes in laboratory and wild mice. To do so we collected data from other laboratory QTL crosses (“panels”) involving the SM/J strain, namely the LG/J × SM/J crosses by the Cheverud and later the Palmer laboratories and the NZB/BINJ × SM/J cross by Stylianou *et al.* 2006, for a total of 4552 mice (Supplementary Table S4; Cheverud 1996; Stylianou *et al.* 2006; Norgard *et al.* 2008; Parker *et al.* 2011). An inherent limitation in many meta-analyses is the variation in protocols and the underlying datasets. We found that among our measurements, 16-week weight was the only trait that was similar enough across the datasets to allow a meta-analysis. We merged the SM/J-polarized autosomal genotypes from the datasets by imputation, yielding a common set of 8671 markers (mostly driven by the most densely genotyped panel LG/J × SM/J F34).

**Figure 5:**
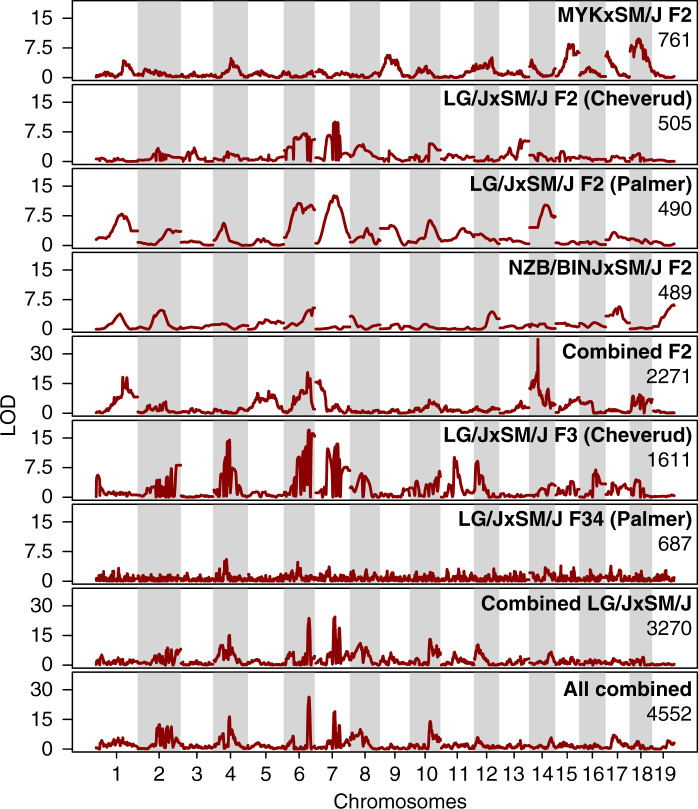
Largely distinct genetic basis of body weight control across a set of crosses sharing an SM/J parent. A meta-analysis was conducted by combining six mapping panels involving the SM/J strain, giving a total sample size of 4552 individuals. The extent of statistical support (LOD) is shown for each panel, as well as groups of panels together, taking their origins into account. Note that the LOD profiles in the F3 and F34 panels appear to fluctuate due to an imputation artifact. This is caused by the much higher density SNP arrays used in these datasets, which can lead to uncertain SM/J allele assignments. This artifact does not affect the main contour of the LOD profile, which is the focus of this analysis. Among the panels, only the MYK × SM/J cross from this study involved wild mice. At the broader QTL level, we did not observe strong overlaps between the Faroese panel and the other panels. Due to the larger size of the various LG/J × SM/J panels, much of the shared signals appear to correspond to panels involving LG/J.

Overall, we observed moderate to little evidence of shared QTLs, even though the panels share a common SM/J parental line (mean Pearson’s correlation = 0.20, Supplementary Figure S4). In principle, QTL sharing may be due to the same haplotype combinations segregating in separate panels. Alternatively, this could be due to different mutations affecting the same genes. Regardless, we found little evidence here to suggest allele-sharing being a major driver behind the QTL signals. This is in contrast to the comparison between the two LG/J × SM/J F2 panels, whose LOD contour track with each other (Figure 5, Pearson’s correlation: 0.85, cf. F2 panels involving MYK: −0.18, −0.05 and −0.08; NZB: −0.18; 0.51 and 0.39; Supplementary Figure S4). In fact, the MYK × SM/J panel is the only one consistently showing a negative correlation with the other panels. This is underscored by the 6 genome-wide significant *bw16w* QTLs, where only *bw16wq1* seem to be shared generally with the other F2 panels. In a full, combined meta-analysis across all data, we observed a strong QTL on Chr14, but in an intermediate, non-overlapping position between *bw16wq14* and the telomeric QTL from the Palmer LG/JxSM/J F2 panel (Figure 5—“All combined”).

Given the lack of clear overlap across the various F2 panels, it was perhaps notable that mapping using all F2 samples (*N* = 2271) seem to show some surprising interactions between the panels. Whereas the QTLs on Chr1 and Chr6 seem to benefit from pooling, the suggestive overlapping QTL on Chr4 now appear to have little to no statistical support (“Combined F2”, Figure 5). Taken at face value, it would support the interpretation that MYK and LG/J carry distinct alleles on Chr4, despite the appearance of overlapping QTL. On Chr14, there is now a striking QTL that appears to overlap a peak from the Palmer F2 panel, but the signal from other individual F2 panel would appear to be unremarkable. Part of the challenge in interpreting the combined mapping would stem from the mixed coding of all non-SM/J alleles together, *e.g.*, lumping MYK together with LG/J, which would cause poor fits of the QTL model when in fact multiple allelic effects exist.

Outside of the F2 panels, our results follow broadly established patterns: LOD profiles are sharper in advanced intercrosses (F3 and F34 panels, Figure 4), implying increased genetic resolution, but possibly at the cost of relatively decreased mapping power (originally discussed in Parker *et al.* 2014). Combined mapping here shows a clear genetic signal, however, since most of the panels are made up of LG/J × SM/J progenies, the LOD profile from the combined mapping appear to be driven largely by that genetic background (Figure 5, bottom two panels, *N* = 3270 and 4552, respectively).

Overall, our results show that the data do not support the assumption of a broad sharing in the genetic basis of body weight changes between the laboratory and in the wild, at least among the comparisons we have attempted here. Whether using a formal QTL analysis like above, or by cross-referencing genomic intervals and their overlap with the parallel selected regions found across body weight selected laboratory lines (Chan *et al.* 2012), we did not detect a correlation. Additionally, comparison with the previous example of island gigantism on Gough Island also showed almost complete non-overlap in 16 total QTLs, even though the two studies together detected QTLs on 15 out of 20 chromosomes (with the lone exception of the QTLs on Chr9 *bw8wq9*, *bw14wq9*, and *bw16wq9*) (Gray *et al.* 2015). In that sense, the Faroese mouse seem to have largely followed its own unique genetic trajectory, and the laboratory and natural examples of outwardly similar changes represent largely distinct sampling of available genetic variation.

## Discussion

The house mice on the Faroe Islands belong to the remarkable examples of rapid evolution among wild mice on islands (Berry *et al.* 1978a, 1978b). Since their scientific discovery and description more than a century ago, the Faroese mice have attracted considerable interest in their large size and their unique phenotypes (Eagle Clarke 1904; Degerbøl 1942; Berry *et al.* 1978a). Berry and colleagues have first noted a great heterogeneity between the islands in both morphological features and allozyme diversity, and hypothesized that colonization bottlenecks as well as selection combined to cause rapid evolution within a span of only two to three hundred years (Berry *et al.* 1978a). The molecular analysis has suggested a colonization through the Vikings several hundred years earlier (Chan and Tautz, unpublished results and Jones *et al.* 2011), with an origin of the mice from south-western Norway, or possibly Denmark/Northern Germany (Jones *et al.* 2011). These are areas of admixture between *M. m. domesticus* and *M. m. musculus* and *M. m. musculus* markers can indeed also be found in the Faroese mice (Jones *et al.* 2011). Hence, genetic background of these populations is likely rather heterogeneous and differ between the individual Faroese islands.

Here we conducted a genetic mapping experiment between a large island mouse from one of the Faroe Islands (Mykines/MYK) and a laboratory strain (SM/J). This latter strain represents on average smaller mice and has been used before for mapping crosses, which allowed us to compare directly with previous laboratory mapping results [a comparable mapping for the large mice from Gough island used a different reference strain background (WSB) (Gray *et al.* 2015)]. We will discuss here various factors that can contribute to the increased sizes in these mice: environmental *vs* genetic components; background/parent-of-origin genetic effects *vs* mapped genetic effects; sources of such variations; and the implications of our results on the genetic architecture underlying laboratory and natural selection response.

Interestingly, in both the Gough island and the Faroe island study, only the wild caught mice from these islands are unusually large. When brought into the laboratory conditions, their weight range is closer to other wild-derived strains kept under laboratory conditions, although they are still heavier and longer than the reference strains used for mapping. Still, this observation implies that non-genetic mechanisms (*e.g.*, plasticity or microbiome) contribute to the phenomenon of large mice on islands. Given this rather strong environmental effect, it should be worthwhile to explore this further in dedicated experiments.

Our mapping panel allowed us to recover 111 QTLs corresponding to different types of traits, including body weight, length, liver weight and growth hormone levels. Among our QTLs, we found a stronger trend toward finding QTLs for later growth. In fact, for our single trait QTL analysis, the one trait with no detected QTL was body weight at 4 weeks, presumably due to a limited sample size (199 measurements, *vs* an average of 527 measurements for the rest). This is in contrast to the Gough Island study, where the authors found greater relevance in earlier growth phases (Gray *et al.* 2015). Interestingly, by partitioning our data according to cross direction, we have found support for a MYK parent-of-origin effect contributing to greater overall growth by week 16, despite starting with slightly lower weaning weights (Supplementary Figure S2). This genetic signal, however, must be balanced against other possible parent-of-origin or maternal effects, which we would not be able to disentangle from our cross design (see Hager *et al.* 2008).

Alternatively, the *musculus* hybridization event may have contributed to a unique genetic makeup in the Mykines compared to the Gough mice, which are of pure *M. m. domesticus* origin. There is now increasing evidence from many natural systems that such events can help create the conditions for novel phenotypes and adaptations and in extreme cases, speciation (Mallet 2007; Nolte and Tautz 2010; Song *et al.* 2011; Heliconius Genome Consortium 2012; Huerta-Sánchez *et al.* 2014; Sankararaman *et al.* 2014; Linnenbrink *et al.* 2020). One tentative datapoint in support of hybridization as a source of possibly composite QTLs (here, putatively of *M. m. musculus* origin) would be a somewhat larger effect size at the body weight QTLs than those reported in the Gough Island or even in the LG/J × SM/J crosses (Cheverud 1996; Norgard *et al.* 2008; Gray *et al.* 2015) (but see QTLs from divergent selection lines, *e.g.*, BEH × BEL) (Brockmann *et al.* 2004). This is despite the larger difference in the body weight between the Gough Island mice and WSB compared to MYK–SM/J here (and accordingly, the MYK allele increases body weight only in six out of ten weight QTLs, or 65% overall). Additionally, we were able to explain a substantial proportion of variation in tail length, a trait known to differ between *musculus* and *domesticus* mice.

###  

#### Polygenic architecture and parallel selection

The use of the common laboratory small strain SM/J made it possible to directly compare against previous laboratory panels sharing the same strain. Remarkably, there is only minimal evidence of QTL sharing between these different studies. This can be interpreted as evidence for a highly polygenic architecture. For studies in human traits, especially for height (body size), it has become clear that a substantial portion of genetic contribution comes from variants scattered across the entire genome, whose effect would lie below detection thresholds (Yang *et al.* 2010), which has led to the formulation of the omnigenic model (Boyle *et al.* 2017). If almost any gene can contribute to a quantitative trait, one has to conclude that given QTLs in mapping experiments reflect more the presence of functional polymorphisms in a given genetic background, rather than the relative importance of the genes for the given trait. Hence, the rather complex genomic background of the MYK mice, with an admixture from *M. m. musculus,* is expected to yield different major associations. We have previously shown this effect also for the mapping of skull shapes in pure *M. m. domesticus* genomic backgrounds, versus mice from a hybrid zone (Pallares *et al.* 2014, 2015, 2016). But we note that even among other panels derived from laboratory strains of mostly *M. m. domesticus* origin, we still detect few overlapping QTLs (Figure 5, n.b. there is however correlation from 0.4–0.6, see Supplementary Figure S4), implying that the genetic background is also crucial for the within-species mappings, as it has also been shown for testing the phenotypic effects of induced mutations (Chandler *et al.* 2013; Sittig *et al.* 2016).

This lack of QTL overlap is in stark contrast to the observation of parallel evolution of the size of mice on several islands. It is also in contrast to the success of finding evidence for QTL overlaps in parallel adapted populations of sticklebacks (Conte *et al.* 2015; Peichel and Marques 2017), as well as in parallel selected lines of mice (Chan *et al.* 2012). In the latter study, we have even found evidence for selective sweeps in Faroe mice, covering loci of two major body size QTLs, albeit from a different island (Sandøy). Close inspection into the actual haplotypes showed that Sandøy mice differ from Mykines at one of the two loci (*Gpr133*), not to mention their distinct composition elsewhere in the genomic background. This may explain why these two major, well-confirmed loci do not show up as QTLs in the mapping with the MYK mice. This example underscores our broader point, that while many remarkable examples of parallel evolution exist (including ones we have published ourselves), a broad-based examination of the data may reveal that a polygenic genetic architecture is far more typical. Hence, any study on parallel selection needs to take into account that the same set of loci could only be revealed if they share the selected variants, either as standing variants among the founding populations or via subsequent gene flow.

## Conclusion

We report here a first study on the genetic basis of the remarkable Faroese house mouse, focusing mainly on body size and weight. Besides their large sizes, these mice were also notable for several unusual morphological characters, especially in the skull. This will be the focus of future studies. Although we have made some progress in understanding the variation among the mice in the Faroe Islands, our work focusing on the Mykines population represents only a small part of the overall picture in the Faroe Islands. Further work, such as admixture mapping from more diverse populations like Sandøy may benefit from the higher genetic resolution beyond those achievable in the F2 cross here.

Besides the Faroe mice, we also expect the current study and others following it to help uncover the genetic underpinning of broader morphological variation in island mouse populations. Indeed, Berry and coworkers have highlighted the broad genetic and phenotypic similarity among Northern Atlantic island mouse populations (Berry *et al.* 1978a). Rather than being completely unique in their various characteristics, they stated that it would be more appropriate to describe the Faroese mice as a unique *combination* of characters. Given broad interests in islands in evolutionary biology and the findings already uncovered by this and previous studies on other islands (Gray *et al.* 2015; Parmenter *et al.* 2016), we are hopeful that further work in these unusual mice will continue to yield useful results to improve our understanding on the principles governing novel adaptations.
